# Attention to renal involvement: report of 17 Joubert syndrome cases in children of a single center in China

**DOI:** 10.1186/s12887-022-03496-8

**Published:** 2022-07-20

**Authors:** Liang Ying, Wang Hui, FuQian, Zhou Nan, Jiang Yeping, Mi Lan

**Affiliations:** Department 2 of Nephrology, Beijing Children′s Hospital Affiliated to Capital Medical University, Beijing Key Laboratory for Chronic Renal Disease and Blood Purification, Key Laboratory of Major Diseases in Children, National Center for Children’s Health, Beijing, 100045 China

**Keywords:** Children, Joubert syndrome, Kidney, RPGRIP1L gene

## Abstract

**Background:**

Joubert Syndrome (JS) is a rare genetic developmental disorder. We are aiming for increasing awareness of this disease especially kidney involvement in children with JS.

**Methods:**

Clinical and genetic data of 17 cases of JS in Beijing children’s hospital in the past 21 years were collected retrospectively.

**Results:**

Twelve males and 5 females, aged from 12d to 15y8m. The most common involvement was neurological system involvement. The second most common involvement was renal involvement: end stage kidney disease in 6 cases (35%), hematuria in 5 cases (29%), proteinuria in 5 cases (29%), renal diffuse lesions in 4 cases (24%), renal cystic lesions in 2 cases (12%), and echogenic enhancement of parenchyma in 2 cases (12%). 10 cases did genetic tests. 3 cases with renal deficiency all had RPGRIP1L gene mutation.

**Conclusions:**

The most common involvement of JS is neurological involvement, and the second is renal involvement. Pediatricians should improve awareness of JS and conduct systemic evaluation of children. More attention should be paid to renal involvement which may be onset hidden but fatal. Early recognition and diagnosis are the goals to delay the start to dialysis and improve quality of patients’ life. The RPGRIP1L gene mutation maybe the most common gene mutation in JS and may have correlations with renal involvement.

## Introduction

Joubert Syndrome (JS; OMIM PS213300) is a rare genetic developmental disorder. It is a predominantly autosomal recessive ciliopathy condition characterized by a distinctive cerebellar and brainstem defect in brain MRI known as the “molar tooth sign” (midline cerebellar vermis hypoplasia, deepened interpeduncular fossa, and thick, elongated superior cerebellar peduncles) because of its resemblance to the cross-section of a tooth on axial imaging [1, 2]. It was first proposed by Marie Joubert in 1969 [3]. The clinical manifestations of JS mainly include neurological system involvement, such as dystonia, ataxia, backward development, intellectual disability, eye movement disorder, abnormal respiratory rhythm, etc. It may also involve multiple systems such as kidney, retina, liver and bone. At present, JS in children are mainly reported on individual cases in China, but large sample survey lacks. In this study, we retrospectively collected clinical data of children diagnosed with Joubert syndrome in national center for children’s health, Beijing children’s hospital from January 2000 to January 2021 for comprehensive evaluation and discussion, aiming to improve the understanding of this disease.

## Patients and method

### Patients

Patients matching the JS diagnostic criteria in Beijing children’s hospital from January 2000 to January 2021 were included in the study. Diagnostic criteria of classic JS include: 1. Cranial magnetic resonance imaging (MRI) findings demonstrating the “molar tooth sign” (MTS) on axial imaging with three components: midline cerebellar vermis hypoplasia, deepened interpeduncular fossa, and thick, elongated superior cerebellar peduncles, 2. Hypotonia in infancy, 3. Developmental delay/intellectual disability of variable severity, 4. One or both of the following (not absolutely required but supportive of the diagnosis): a. Irregular breathing pattern in infancy (episodic tachypnea and/or apnea), b. Abnormal eye movements (including nystagmus, jerky eye movements, and oculomotor apraxia or difficulty with smooth visual pursuits) [1].

## Methods

### Clinical data collection

Clinical data such as medical histories, physical examinations, clinical manifestation, relevant laboratory results, imaging results, genetic tests results were collected through the hospital electronic medical record system. Outcomes and follow-up data were obtained through telephone follow-up. The follow-up period ranged from 11 months to 156 months.

### Statistical analysis

We statistically analyzed all data using SPSS software version 25.0 (IBM Corp., Armonk, NY, USA). Continuous variables are presented as mean with standard deviation (mean ± SD) or median with interquartile range (IQR). Categorical variables are presented as frequencies and percentages (n (%)).

## Results

There are 17 patients matching the JS diagnostic criteria in Beijing children’s hospital from January 2000 to January 2021. The clinical data of 17 patients are summarized in Tables 1, 2 and 3.

### Basic information of 17 cases

There were 12 males and 5 females and the male-female ratio was 2.4:1. The age ranged from 12 days to 15y8m, among which 2 cases (11.8%) were newborns, 10 cases (58.8%) were 29 days to 3 years old, 2 cases (11.8%) were 3–10 years old, and 3 cases (17.6%) were over 10 years old. Department included: nephrology department, neonatal department, respiratory department, neurological department and intensive care unit.

One case were hospitalized 3 times, four cases were hospitalized 2 times, and twelve cases were hospitalized once. The time from onset to be hospitalized ranged from 5 days to 4 years, and the average time from onset to visit was 34 months. All the basic information were concluded in Table 1.

**Table 1:** Basic information of 17 cases

Variable	No	%^a^
**Age at onset**		
Range (mean)	12d-15y8m(3y11 m)	
Newborns	2	11.8
29 days to 3 years old	10	58.8
3 years old to 10 years old	2	11.8
Over 10 years old	3	17.6
**Sex**		
Male	12	70.6
Female	5	29.4
**Time from onset to be hospitalized**		
Range (mean)	12d-4.5y(34 m)	
**Department**		
Nephrology department	5	29.4
Neonatal department	2	11.8
Respiratory department	3	17.6
Neurological department	5	29.4
Intensive care unit	2	11.8
**Repeated hospitalization**		
once	12	70.6
2 times	4	23.6
3 times	1	5.8

^a^Percentages are calculated by individual for all variables

### Personal history

Six of 17 cases had positive personal history, details shown in Table 2. And all the patients were of Han descent.

**Table 2:** Personal history

Patient No.	Personal history
1	Diagnosed with “floating cranial disease” after birth
2	Diagnosed with“neonatal pneumonia, neonatal respiratory distress syndrome, neonatal hypoxic ischemic encephalopathy, hyperbilirubinemia, congenital laryngeal chondroplasia, pulmonary hypertension. Bronchopulmonary dysplasia?”
3	Cerebellar vermis loss in ultrasound when at 24 weeks of gestation
7	Underwent “congenital cleft palate repair” at 4 years old
8	Left eyelid ptosis after birth
9	Underwent “bimanual polydactylotomy” surgery at 1 years old

### Systemic involvement of 17 cases

The incidence of neurological system involvement: mental retardation in 7 cases (41%), muscle strength, dystonia and convulsion in 4 cases (24%), and apnea in 2 cases (12%). The incidence of renal involvement: end stage kidney disease in 6 cases (35%), hematuria in 5 cases (29%), proteinuria in 5 cases (29%), renal diffuse lesions in 4 cases (24%), renal cystic lesions in 2 cases (12%), and echogenic enhancement in parenchyma in 2 cases (12%). Incidences of systemic involvement and renal involvement are showed in Table 3.

**Table 3:** Systemic involvement of 17 cases

Characteristic	No	%^a^
**Neurological involvement**		
Mental development lags	7	41
Muscle strength and tension decreased	4	24
Convulsions	4	24
Apnea	2	12
**Renal involvement**		
End stage kidney disease (HD/PD^b^)	6(4/2)	35
Hematuria	5	29
Albuminuria	5	29
Diffuse renal disease	4	24
Renal cystic lesions	2	12
Enhanced echogenicity in parenchyma	2	12
**Facial defects**		
Special facialfeatures, straight eyebrows	1	6
Congenital cleft palate	1	6
**Ocular abnormalities**		
Drooping eyelids	2	12
Limited eye movement	2	12
Wide bilateral eye distance, low ear position	1	6
**Limb abnormalities**		
Polydactyly	1	6
Ulnar skin tag on metacarpophalangeal joint	1	6
**Liver involvement**		
Liver function damage	2	12
Liver enlargement, echo thickening	1	6
**other**		
Tongguan Palm	1	6

^a^Percentages are calculated by individual for all variables

^b^HD/PD: Hemodialysis/Peritoneal dialysis

### Typical brain imaging findings

All cases had typical brain imaging findings: vermis hypoplasia, irregular enlargement of the four ventricles, wide and deep sulcus in the upper cerebellum, thickens of the upper cerebellum foot, and molar sign in the midbrain. As shown in Image 1-4.

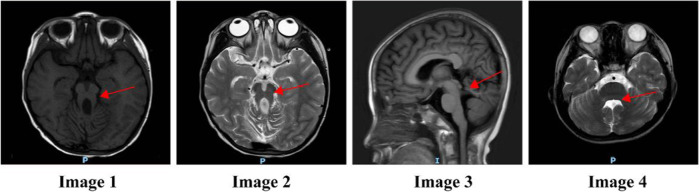



Images 1, 2 Intercrural fossa deepens, upper cerebellar angle lengthens and thickens, showing molar sign (arrow)

Image 3 Thickened upper cerebellar foot, almost perpendicular to brainstem (arrow)

Image 4 Enlarged upper fourth ventricle with batwing sign (arrow); The cerebellar vermis was absent, the cerebellar hemispheres showed midline cleavage sign, and the middle of the fourth ventricle showed triangular sign

### Typical eyemovement disorder

Typical eyemovement disorder showed in [see Additional file 1]. The patient in the video showed horizontal eye movement disorder.

### The gene results of 10 cases

Ten of 17 cases did exon sequencing tests. 3 had RPGRIP1L gene compound heterozygous mutation, 2 had CC2D2A gene compound heterozygous mutation, 1 hadCSPP1 gene compound heterozygous mutation, 1 had C5ORF42 gene compound heterozygous mutation, 1 had CPLANE1 gene compound heterozygous mutation, 1 had ARL13B gene compound heterozygous mutation and 1 had NPHP1 gene homozygous mutation. 3 cases with renal deficiency all had RPGRIP1L gene mutation. The results are summarized in Table 4.

**Table 4:** Genetic results of 17 inpatients with Joubert syndrom (listed in ascending order by patients No., starting with No.1)

Patient No.	Sex	Age	Chromosome location	Gene locus	Gene/locus MIM number	Hom/ Comp het	Nucleotide/amino acid	Inheritance pattern	Phenotype in OMIM	ESRD^a^ HD/PD^b^
5	F	1y8m	Chr16:53652959 Chr16:53692387	RPGRIP1L	610,937	comp het	c.3354G > A/p.W1118X c.1351-11A > G/−	AR	Joubert Syndrome 7	(+)
6	F	5y9m	Chr16:53720364 Chr16:53679824	RPGRIP1L	610,937	comp het	c.757C > T/p.G253T c.2396G > A/p.C799T	AR	Joubert Syndrome 7	(+)
7	M	11y6m	Chr16:53683001 Chr16:53708929	RPGRIP1L	610,937	comp het	c.2179G > A/p.G727S c.882G > T/p.E294D	AR	Joubert Syndrome 3	(+)
8	M	1y6m	Chr8:68026038 Chr8:68024300	CSPP1	611,654	comp het	c.1215-1_1220delinsATTTTGTTTAATTTTGTTTG/− c.1214G > A/ p.A405G	AR	Joubert Syndrome 21	(−)
10	F	7 m	chr4:15552462 chr4:15511824	CC2D2A	612,013	comp het	c.2197G > A/ p.G733A c.501G > T/ p.L167A	AR	Joubert Syndrome 9	(−)
12	M	2y3m	Chr5:37221504 Chr5:37162670	C5ORF42	614,571	comp het	c.2668C > T/ p.G890T c.7589-2A > G/−	AR	Joubert Syndrome 17	(−)
13	M	4y5m	Chr5:37181031 Chr5:37213736	CPLANE1	614,571	comp het	c.5498C > A/p.S1833X c.2845 T > C/p.Y949H	AR	Joubert Syndrome 17	(−)
14	M	11 m	Chr4:15552462 Chr4:15511824	CC2D2A	612,013	comp het	c.3866C > T/p.T1289I c.4206G > A/p.W1402X	AR	Joubert Syndrome 9	(−)
15	M	15y8m	Chr3:93722607 Chr3:93722629	ARL13B	608,922	comp het	c.235C>T/p.R79W c.257A>G/p.Y86C	AR	Joubert Syndrome 8	(−)
17	M	2y2m	Chr2:110917769 Chr2:110917769	NPHP1	607,100	hom	c.1186C>T/p.L396P	AR	Joubert syndrome 4	(+)

^a^ESRD: end stage renal disease; ^b^HD/PD: Hemodialysis/Peritoneal dialysis

### Gene-phenotype correlation in Joubert syndromes

**Fig. 1:**
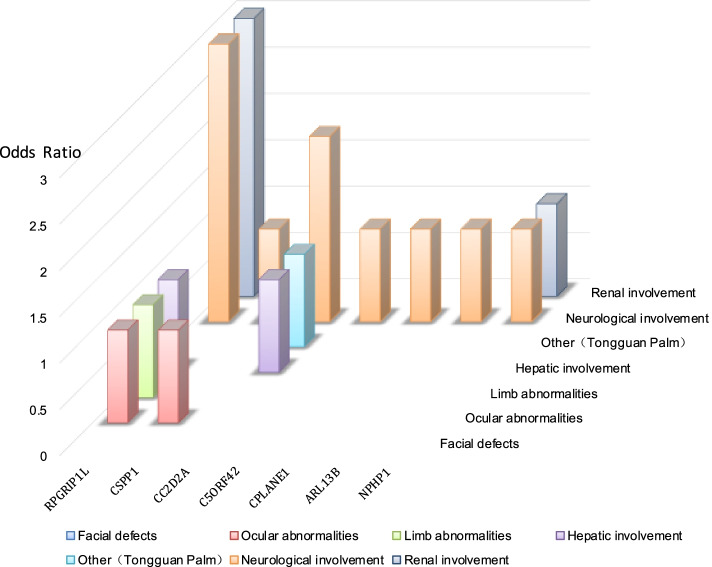
Gene-phenotype correlation in Joubert syndromes

We next examined associations between the clinical features of JS and each genetic cause. The gene-phenotype correlations showed in Fig. 1.

Bar graph indicates each involvement and the more frequently related genes, showing gene-phenotype correlation in Joubert syndrome. The RPGRIP1L gene was strongly correlated with the renal phenotype.

### Follow up

The current outcome of 17 patients was concluded through telephone follow-up, among which 7 patients were lost to follow-up, with a total loss rate of 41.2%. The follow-up period ranged from 11 months to 156 months. Among the 6 JS patients with end stage kidney disease, 2 were lost to follow-up, 1 died of ESRD after 5 years’ hemodialysis when he was 20y, 1 received kidney transplantation, and 2 were still receiving maintenance dialysis (a 7y7 m girl received peritoneal dialysis for 1.7 years, a 13y1m boy received hemodialysis for 1.5 years). The mortality rate of JS children with end stage kidney disease was as high as 16.7%.

## Discussion

Cilia dysfunction can lead to a series of complex and serious diseases, involving multiple systems, including kidney, liver, nervous system, eyes, respiratory system, reproductive system, bone, etc. These diseases are known as ciliopathies. Joubert Syndrome (JS) is a typical ciliopathies caused by disruption of ciliary transition-zone architecture [4].

Joubert syndrome is a rare autosomal recessive genetic disorder although a few X-linked recessive cases have been reported [5]. An estimated incidence of JS is 1/100,000 to 1/8000 [5, 6]. JS is characterized by a dysplasia of the cerebellar worm and a “molar tooth sign” (MTS) on brain magnetic resonance imaging. The disease was first reported by Marie Joubert in 1969, and was first identified in a French-Canadian family in which all four siblings had mental disabilities, ataxia, eye movement disorders, and cerebellar worm dysplasia. Boltshauser and Isler reported three similar cases in 1977 and named the disease Joubert syndrome. However, with the in-depth understanding of the disease and the development of genetests. The injury of other organs besides nervous system plays an increasingly important role in prognosis.

### Neurological involvement is most common

The main clinical manifestations of JS are neurological system involvement like development lag, intellectual disability, dystonia, eye movement loss, ataxia, abnormal respiratory rhythm, etc. In our study, abnormal development of JS central nervous system includes retardation of intellectual development, serious intellectual deficiency and intelligence measured at boundary value. The incidence of neurological involvement was the highest, and nearly half of neurological symptoms was mental retardation, suggesting that mental retardation was the most common manifestation of JS in clinical practice. Another manifestation of the neurological system was ataxia and dystonia. Most children were found to be abnormal when sitting alone or walking alone in infancy. It is of great significance to do brain MRI, intelligence measurement and genetic test for diagnosis of JS in children with mental retardation at an early stage.

Although neurological system involvement is the most common, it progresses relatively slowly and is not the major cause of death. In a cohort of 565 patients with JS (40 of them deceased), deceased status was found to be associated with extra-neurological features instead of neurological features [7].

### Kidney involvement is fatal but easy to be neglected

Nearly 1/3 of JS patients have kidney involvement according to previous reports. Our study suggests that kidney is the second most common JS involved organ after neurological system, and the incidence of end stage kidney disease can reach to 35%(6/17), which is basically consistent with previous research data. Jennifer C. Dempsey did a survey of motality of JS of the approximately 850 families, finding that the most common causes were kidney failure (15/40, 37.5%) and respiratory failure (14/40, 35%). The main cause of death in patients older than 6 years old is renal insufficiency [7].

Previous reports on JS focus more on the evaluation and treatment of neurological system function, but lack sufficient attention to the kidney. As we observed, lots of JS patients never did any tests related to kidney after they were diagnosed. We suspect the reason why we used to pay more attention to neurological system instead of kidney is that there is no diagnosis criteria relevant to kidney. Also, the kidney is known as a “silent” organ, meaning kidney failure is a latent process and can be easily neglected. As we can see, the primary manifestation of 6 patients in our study were end stage kidney disease. 4 of 6 had hemodialysis while 2 had peritoneal dialysis. Patients with end-stage renal failure have to choose kidney transplant or dialysis. Complicaitons of end stage renal failure like heart failure, pulmonary edema, anemia, secondary hyperparathyroidism, metabolic bone disease influence the quality of life badly and increase motality of patients, posing a great economic burden on society and families. Therefore, early renal assessment plays an important role in improving the long-term survival rate of children.

It has been previously reported that renal damage in JS patients can be manifested as renal tubular structure abnormalities and/or cystic lesions of renal cortex medulla oblongata, which may lead to polydipsia, polyuria and renal cystic lesions, and then develop into acute/chronic renal insufficiency, and eventually kidney failure. However, our study found that the incidence of abnormal urinalysis, such as albuminuria (29%) and hematuria (29%), was higher than that of renal cystic lesions (12%), suggesting that noninvasive simple urinalysis can be used as the first choice for renal examination in children suspected of JS, and urological ultrasonography should be completed at the same time. For patients suspected of JS clinically, evaluation of renal function and structure should be carried out as soon as possible. Urine concentrating ability is also an effective means of monitoring for CKD in patients. Urinary concentrating defect was a significantly sign in patients with CKD in an early stage (Nuovo et al., 2018). In a study of 93 patients with JS, CKD was predicted in > 75% with urine osmolality < 600 mOsm/kg. Early detection of renal structure or function damage, early intervention and proper complication assessment may delay the timing of kidney transplantation or dialysis.

Joubert syndrome is a disease with strong clinical heterogeneity. Joubert syndrome-related disorders (JSRD) has neurological signs along with variable multiorgan involvement including skeletal changes such as polydactyly, ocular coloboma, retinal dystrophy, renal disease (cystic dysplasia/nephronophthisis), and hepatic fibrosis. Nowadays JS has been considered as a type of renal ciliopathy. As we found in the study, kidney involvement of JS is very common, but the classical diagnostic criteria of JS has no kidney related criteria. Our research shows that there are 6 cases in children have end stage renal disease, so we should attach importance to JS renal involvement, the kidney related criteria into the diagnostic criteria may be an effective measure.

### Correlation between genotype and phenotype

Joubert syndrome is a model for untangling recessive disorders with extreme genetic heterogeneity [8]. There are a few Joubert syndrome cases with clear genetic diagnosis reported in China, but lack studies on large samples. The 10 genetic results of our study suggested that there were 3 had RPGRIP1L gene compound heterozygous mutation, 2 had CC2D2A gene compound heterozygous mutation, 1 had CSPP1 gene compound heterozygous mutation, 1 had C5orf42 gene compound heterozygous mutation, 1 had CPLANE1 gene compound heterozygous mutation, 1 had ARL13B gene compound heterozygous mutation and 1 had NPHP1 gene homozygous mutation. RPGRIP1L gene mutation is the most common.

It is reported that kidney disease affects up to 1/3 of patients with Joubert syndrome and is more common in those with pathogenic variants in CEP290, TMEM67 and AHI1 genes. According to previous reports, variation of RPGRIP1L gene can lead to Joubert syndrome, but the detection rate is low, only 1–2% [9, 10], mostly accompanied by kidney damage. However, the proportion of RPGRIP1L mutations is the highest in our study of Joubert syndrome. Li also reported a JS Chinese children with RPGRIP1L gene mutation in 2018 [11]. In terms of the correlation between RPGRIP1L mutation and kidney involvement, relevant studies in western countries are quite inconsistent with those in China. We suspect that the differences in genetic mutations is probably related to human race. More research is needed to confirm the reliability of this conclusion and to explore the exact mechanism.

Three patients with RPGRIP1L gene and NPHP1 gene mutation all had end stage renal failure, suggesting that RPGRIP1L gene and NPHP1 gene mutation may be related to kidney involvement especially end stage kidney disease. More attention should be paid to kidney involvement in patients with RPGRIP1L gene and NPHP1 gene mutation. RPGRIP1L gene is also known as NPHP8 gene which is one of genes cause nephronophthisis (NPHP, MIM 256100). NPHP constitutes the most frequent genetic cause of endstage renal disease (ESRD) in children and young adults [12]. In nephronophthisis patients, the genes encoding ciliary device component proteins are mutated, and these proteins are expressed in protocilia, matrix and centrosome. NPHP1 gene mutations are the most common, accounting for about 20% of cases. Other gene mutations include NPHP2–13 genes. NPHP-8/RPGRIP1L plays an important role in cilia formation and cilia-mediated chemosensation in a cell type-specific manner. RPGRIP1L was located within the deletion of the ft.^−/−^ mouse led to the discovery of RPGRIP1L mutations as the cause of NPHP type 8 [13]. A study analyzed a cohort of 56 patients from central Europe and the United States with NPHP and identifed four different mutations in the RPGRIP1L gene in five different families [14]. In the case of RPGRIP1L, a clear genotype-phenotype correlation seems to exist, with only one truncating mutation or a homozygous missense mutation causing NPHP, whereas homozygous truncating mutations cause Joubert syndrome related disorders [15].

Our report supports the notion that genotype-phenotype correlations may help guide individualized management in JS patients. More further basic researches are needed to explore possible associations between systemic involvement and genetic causes.

### Pay attention to systemic involvement

Abnormal eye movement is the most characteristic and frequently occurring developmental abnormality in JS patients, which is specifically manifested as absence of follow-up and disappearance of vestibular eyeball reflex. Nystagmus is also common, and may also be accompanied by strabismus and ptosis. Our study found that JS ocular abnormalities including eye movement disorder, drooping eyelids, too wide eye spacing accounted for 30%, lower than the previous related data. Ophthalmic examination should be performed routinely to improve the rate of early diagnosis of ocular abnormalities. Other ocular defects include lesions of the retinal pigment epithelium, choroid, and sensory nerves. Lesions of the eye are usually screened and evaluated by visual acuity, fundus, and electro retinogram.

A small number of JS patients will have liver lesions, mostly in the form of liver fibrosis, which is specifically manifested as abnormal liver-related enzymes in the serum and gradually manifested as hepatosenomegaly, portal hypertension and esophageal varices, and eventually develops into liver fibrosis. In our study, there were 2 cases with abnormal liver enzymes (12%) and 1 case with abnormal liver structure (thickening of echo) (6%), which were significantly less than the cases with nervous system and kidney involvement.

Some JS patients due to skeletal dysplasia refers to toe deformity, scoliosis, some patients have abnormal characteristics of maxillofacial, such as encephalocele craniocerebral deformity, mouth facial midline defects (cleft lip and cleft palate, paging tongue, etc.), 1 patient had “congenital cleft palate repair” surgery when 4 years old, 1 patient had polydactyly and do the resection surgery when 9 years old. So when we see patients who have skeletal dysplasia or polydactyly and kidney injury should take nephronophthisis including JS into consideration. It is an obvious sign.

JS is a systemic disease and should be evaluated systemically as soon as possible when a patient is clinically suspected to have this disease. It is necessary to improve the pediatrician’s awareness of the disease to provide a more comprehensive assessment of patients. Wesuggestto attach importance to JS renal involvement. Adding the kidney related criteria into the diagnostic criteria may be an effective measure to recognize it.

### The prognosis

There is no unified data on the specific mortality of JS. In our study, 1 of 17 patient died, the motality rate is 5.9%. But there is statistical error due to the limited sample capacity.

The average age of death in children with Joubert syndrome is around 7 years, with renal insufficiency being the main cause of death in patients older than 6 years [7]. We found the only decreased patient in our study died of end stage kidney disease. It is consistent with previous studies suggesting that renal involvement is a high risk factor for death in children with JS.

## Conclusions

With the rapid development of medical technology and gene detection methods in recent years, though Joubert syndrome is a rare disease, the detection rate is increasing year by year. In addition to the nervous system, kidney is the main affected organ of JS. More attention should be paid to kidney involvement. We suggest to attach importance to JS renal involvement. Adding the kidney related criteria into the diagnostic criteria may be an effective measure to recognize it more earlily. Early identification of kidney damage in children with JS, appropriate intervention and monitoring are helpful to improve the quality of life and survival years of children. Patients should be monitored for signs and symptoms of CKD including blood pressure measurements and serum creatinine, blood urea nitrogen (BUN), and blood count with hematocrit/hemoglobin [16]. Gene results help us predict the prognosis of the disease due to a certain correlation between genes and phenotypes.

## Data Availability

The datasets used and/or analysed during the current study are available from the corresponding author on reasonable request.
